# Trends and disparities in osteoarthritis prevalence among US adults, 2005–2018

**DOI:** 10.1038/s41598-021-01339-7

**Published:** 2021-11-08

**Authors:** Yingke Xu, Qing Wu

**Affiliations:** 1 Department of Epidemiology and Biostatistics, School of Public Health, University of Nevada, Las Vegas, Nevada 89154 USA; 2 Nevada Institute of Personalized Medicine, College of Sciences, University of Nevada, Las Vegas, Nevada 89154 USA

**Keywords:** Epidemiology, Osteoarthritis

## Abstract

Studies reporting trends and disparities of osteoarthritis (OA) in the United States are limited. We aimed to examine trends and disparities of OA prevalence among US adults, from 2005 to 2018. Continuous National Health and Nutrition Examination Survey (NHANES) data from 2005–2006 to 2017–2018 were analyzed. Age-adjusted and self-reported OA prevalence, stratified by race/ethnicity and socioeconomic status (SES), was calculated separately for men and women. The linear trend and the association between the survey cycles and OA prevalence were assessed. Age-adjusted and self-reported OA prevalence linearly increased in the seven survey cycles (both P_linear trend_ ≤ 0.0002) in men and women. Non-Hispanic Caucasians (both P_linear trend_ ≤ 0.0001) in both genders and Non-Hispanic African Americans women (P_linear trend_ ≤ 0.0001) had significantly increasing linear trends in OA prevalence. In addition, people with lower SES had a lower age-adjusted prevalence of self-reported OA when compared to those with higher SES. The increasing linear trends still existed among both men and women after adjusting for multiple confounders (both P_linear trend_ ≤ 0.002). There were significant rising trends and disparities in self-reported OA prevalence among US men and women between 2005 and 2018.

## Introduction

Osteoarthritis (OA) is a slowly progressive disease that affects joint systems in humans^1^. This disease negatively influences millions of individuals worldwide and is a major cause of pain, comorbidity, and mortality^2^. Typically, people with OA will experience lower employment than those without OA since the disease is a leading cause of disability^3^. OA is the most costly condition for privately insured patients in this country, accounting for over $6.3 billion in healthcare expenses^4^. The number of US adults with arthritis is expected to reach 78 million in 2040^5^. As the most common form of arthritis, OA is associated with an increased economic burden for both individuals and the healthcare system due to its high prevalence^6^.

A limited number of studies have explored the trends and disparities in OA prevalence among US adults^7–9^. Dr. Park et al. reported that OA’s overall prevalence had doubled from 1999 to 2014^7^. Another study suggested that Non-Hispanic African Americans had significantly greater knee OA odds than Non-Hispanic Caucasians during 1991–1994^9^. However, OA’s prevalence trends in the US adult population and within gender and socioeconomic status (SES) subgroups remain unknown after 2014. Notably, the trend in multivariable-adjusted OA prevalence among US adults since then has not been reported in any existing literature.

Therefore, we aimed to examine the trend of OA prevalence in men and women and within race/ethnicity and SES groups from 2005 to 2018. Our study not only included new data after 2014, but we also examined sex-specific trends in OA prevalence during 2005–2018, after adjusting for race, SES, and related risk factors. Our findings will provide a more comprehensive understanding of recent OA trends and disparities in US adults.

## Methods

### Study design

Data from 7 discrete 2-year cycles (2005–2006 through 2017–2018) of the continuous National Health and Nutrition Examination Survey (NHANES) were used to examine the trends of OA prevalence in US adults. NHANES is a nationally representative survey for evaluating the US population’s health and nutrition status at defined periods of time. The plan of operation and sampling scheme are extensively described elsewhere^10^. But briefly stated, the Centers for Disease Control and Prevention employs an intricate, multistage probability sampling design for examining a nationally representative sample across the country every 2 years^10,11^. The data is collected through home interviews and physical examinations. NHANES interviews contain information about demographic, socioeconomic, dietary, and health-related parameters. The physical examination includes medical, dental, and physiological measurements; the detailed methodology and protocols have also been described elsewhere^12^. To produce reliable statistics, NHANES oversamples persons 60 and older who are of African American and Hispanic ethnicity. NHANES study protocol has been approved by the National Center for Health Statistics Research Ethics Review Board. Written informed consent was obtained for all adult participants. All research was performed in accordance with the Declaration of Helsinki. Sample weights in NHANES have been constructed to adjust for non-response, oversampling, and non-coverage. Because of the thoroughness of its research methodology, NHANES data have been widely used over the years to reliably assess many diseases’ prevalence and risk factors. NHANES only collects osteoarthritis information among adults aged 20 or older, which consists of the analytic population in the current study.

### Variables

Since self-reported, doctor-diagnosed arthritis is the most commonly used case definition for prevalence and other epidemiological studies^13–15^. Each NAHNES participant was defined as having OA if he/she answered: “yes” to the question “Has a doctor or other health professional ever told you that you had arthritis?” and “osteoarthritis” to the question “Which type of arthritis was it?” Demographic variables, including age, gender, and race/ethnicity, were ascertained by questionnaire. For the race/ethnicity groups, Mexican American and Other Hispanic were merged into Hispanic, and the remaining groups were Non-Hispanic Caucasian, Non-Hispanic African American, and Non-Hispanic Other, respectively. The educational attainment and family poverty income ratio (PIR) of participants were chosen as SES indicators. Educational attainment was categorized as less than high school, high school graduate/GED, some college, and college graduate or above^16^. PIR was computed as a ratio of the mid-point of the observed family-income category to the family’s appropriate poverty threshold in a given calendar year, as set by the US Census Bureau^17^. Individuals were stratified into three levels based on their PIR: PIR < 1.3 (low income), 1.3 ≤ PIR < 3.5 (middle income), and ≥ 3.5 (high income)^18^. The cutoff point for participating in the Supplemental Nutrition Assistance Program is PIR = 1.3, so individuals with PIR < 1.3 were classified to the low-income group; PIR ≥ 3.5 provides relatively equal sample sizes for each of the three income groups^18^, thus people with PIR ≥ 3.5 were classified to high-income group and those with 1.3 ≤ PIR < 3.5 were the middle-income group. OA-related risk factors were considered and selected based on existing literature and on availability in the NHANES data. Weight status^19^, smoking status^20^, and physical activity^21^ were included in the current study. Participants were categorized as obese if body mass index (BMI-weight in kilograms divided by height in meters squared) was greater than 30^22,23^. Smoking status was categorized into current smokers, former smokers, and non-smokers^24^. Current smokers were respondents who had smoked at least 100 cigarettes during their lifetime and reported smoking either every day or some days at the time of the interview. Former smokers were those who reported smoking 100 cigarettes during their lifetime but currently did not smoke. Otherwise, participants were classified as non-current-smokers. Physical activity was categorized as inactive and active. Participants who were sedentary or only did basic activities, which refers to the light-intensity activities like standing and walking slowly, were considered to be inactive; otherwise, the individuals were classified as active^25^.

### Statistical analyses

Sampling weight was used to account for the complex survey design (e.g., unequal probabilities of selection) during analysis. Estimates were age-adjusted by the direct method to the 2000 US Census population^26^. Age-adjusted OA prevalence in every survey cycle was estimated by race/ethnicity, education level, and PIR level for each gender. Standard errors, which were employed to construct confidence intervals, were estimated using Taylor series linearization. Testing for a difference of age-adjusted prevalence between groups was done using the pairwise t-test. Linear trends during the seven survey cycles were assessed by gender, race, and SES using orthogonal polynomial contrasts. JoinPoint Software (National Cancer Institute, Bethesda, MD) was utilized to determine the slopes and find the inflection point and differences in slopes between the two survey cycles by using piecewise linear regression^27^. If at least one significant change point was found, we report the year the trend shifted; otherwise, we only report P for linear trend. The survey cycle was used as a categorical variable in the analysis. OA prevalence was modeled as a function of the survey cycle after first adjusting for age and then with further adjustments for age, race, educational attainment, and PIR. We performed the analysis using the completed data, and missing data were excluded from the study. Since all variables had < 10% missing data, using complete data is unlikely to cause a biased estimate. Data analysis was conducted using procedure PROC SURVEY of SAS 9.4 (SAS Institute, Cary, NC, USA).

## Results

### Characteristics of the analytic sample

A total of 34,171 eligible participants in NHANES from 2005–2006 to 2017–2018 were included for the analysis; 11.03% of them had OA. The weighted characteristics of participants are presented in Table 1. From 2005–2006 to 2017–2018, the mean (SD) age of participants increased from 46.20 (0.75) years to 48.10 (0.65) years. Additionally, the proportion of Hispanics increased from 11.05 to 14.48%, whereas the percentage of Non-Hispanic Caucasians decreased from 72.13 to 64.27%. The percentage of people having less than a high school diploma decreased during 2005–2018, while the percentage of participants who graduated from college or above increased. The distribution of risk factors of OA for men and women is shown in Supplementary Table 1.

**Table 1:** Weighted characteristics of participants in seven National Health and Nutrition Examination Surveys from 2005 to 2018.

	2005–2006 (N = 4459)	2007–2008 (N = 5084)	2009–2010 (N = 5399)	2011–2012 (N = 4801)	2013–2014 (N = 5094)	2015–2016 (N = 4846)	2017–2018 (N = 4488)
Age, mean (SD) (years)	46.20 (0.75)	46.51 (0.43)	46.84 (0.51)	47.04 (0.88)	47.38 (0.38)	47.59 (0.57)	48.10 (0.65)
Women, No. (weighted %)	2326 (51.94)	2585 (51.82)	2785 (51.96)	2442 (51.82)	2661 (51.82)	2518 (52.17)	2320 (51.77)
**Race, No. (weighted %)**							
Hispanic^a^	1012 (11.05)	1388 (12.73)	1439 (12.72)	944 (13.90)	1072 (13.89)	1447 (14.69)	954 (14.48)
NH-Caucasian	2240 (72.13)	2436 (70.23)	2691 (69.54)	1822 (67.47)	2240(66.75)	1647 (65.16)	1637 (64.27)
NH-African American	1026 (11.51)	1052 (11.04)	975 (11.11)	1243 (11.08)	1048 (11.36)	1010 (11.00)	1019 (10.83)
NH-other	181(5.31)	208 (6.00)	294 (6.63)	792 (7.55)	734 (8.00)	742 (9.15)	878 (10.42)
**Education level, No. (weighted %)**							
< High school	1205 (17.28)	1545 (20.16)	1474 (18.31)	1082 (15.80)	1049 (14.66)	1099 (13.76)	829 (10.40)
High school graduate/GED	1065 (24.92)	1250 (25.07)	1242 (22.79)	1002 (19.87)	1141 (21.74)	1058 (20.72)	1081 (27.25)
Some college	1283 (31.40)	1318 (29.01)	1544 (30.52)	1470 (32.32)	1601 (33.12)	1459 (32.60)	1480 (31.22)
≥ College	906 (26.40)	971 (25.76)	1139 (28.38)	1247 (32.01)	1303 (30.48)	1230 (32.92)	1098 (31.13)
**PIR, No. (weighted %)**							
< 1.3	1166 (17.18)	1551 (20.50)	1813 (21.68)	1724 (24.85)	1759 (24.79)	1567 (21.00)	1274 (20.06)
1.3–3.5	1757 (37.85)	1979 (35.01)	2023 (36.49)	1629 (34.08 )	1749 (34.34)	1924 (36.78)	1855 (35.92)
≥ 3.5	1536 (44.97)	1554 (44.49)	1561 (41.82)	1448 (41.07)	1586 (40.87)	1355 (42.21)	1359 (44.02)

*NH-Caucasian* Non-Hispanic Caucasian, *NH-African American* Non-Hispanic African American, *NH-Other* Non-Hispanic other, *GED* General Educational Development, *PIR* poverty income ratio.

^a^Hispanic includes Mexican American and other Hispanic.

### OA prevalence trends by gender

**Figure 1:**
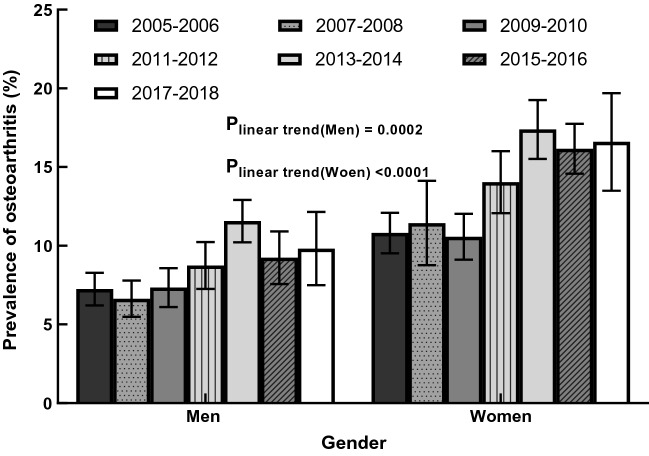
Age-adjusted prevalence of osteoarthritis by gender, 2005–2006 through 2017–2018.

The gender-specific and age-adjusted prevalence of self-reported OA in 2005–2018 appears in Fig. 1. Overall, women had a significantly higher age-adjusted OA prevalence than men (P-value < 0.0001). OA’s age-adjusted prevalence among men increased from 7.25 (95% CI 6.21–8.28%) to 11.56% (95% CI 10.22–12.90%) in 2005-2014, and then decreased in 2015-2018. For women, the age-adjusted OA prevalence increased from 10.81% (95% CI 9.51–12.09%) to 17.39% (95% CI 15.52–19.26%) during 2005–2014, then decreased a little bit and then remained stable. A significant overall linear trend was observed for both men and women (all P_linear trend_ ≤ 0.0002). The Joinpoint analysis and piecewise regression analysis found no inflection point during 2005–2018 for both men (slope = 1.06, P = 0.02) and women (slope = 1.03, P-value = 0.02).

### OA prevalence by race in both genders

**Figure 2:**
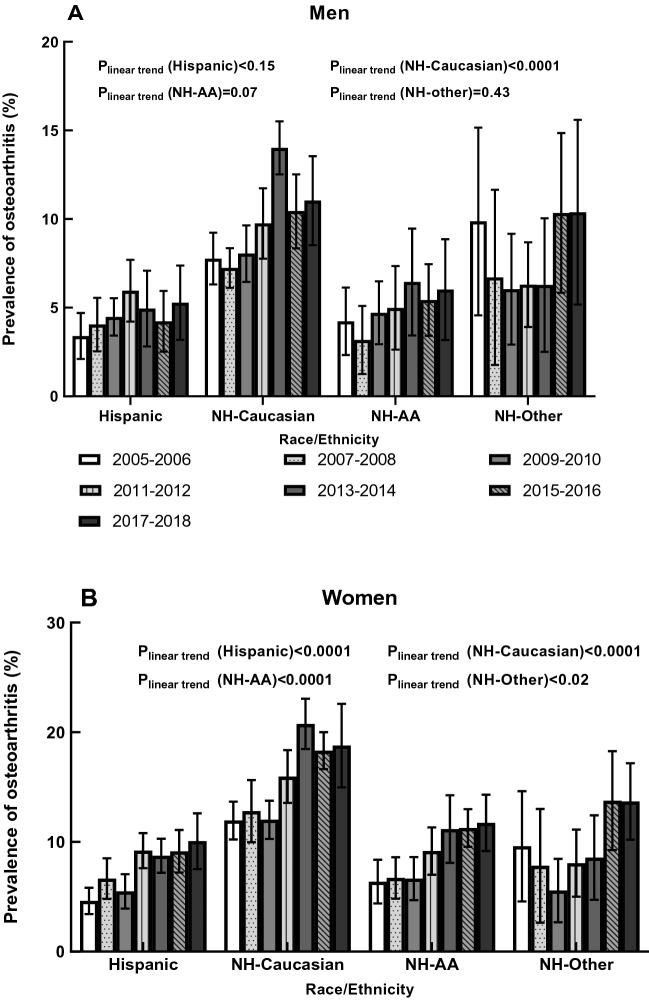
Age-adjusted prevalence of osteoarthritis by race in men and women, 2005–2006 through 2017–2018.

The age-adjusted self-reported OA prevalence by race/ethnicity in men and women is presented in Fig. 2. Non-Hispanic Caucasian men had a higher age-adjusted prevalence of OA than men from the Hispanic and Non-Hispanic African American groups (both P-values < 0.0001). In comparison, Non-Hispanic women had a significantly higher OA prevalence than women in the other three race/ethnicity groups (all P-values < 0.0001). In men, OA’s age-adjusted prevalence among Non-Hispanic Caucasians increased from 7.77% (95% CI 6.31–9.23%) to 14.01% (95% CI 12.52–15.51%) during 2005–2014. Then it decreased to 10.45% (95% CI 8.35–12.52%) in 2015–2016 and remained in a steady state. In addition, a significant linear trend was observed among the Non-Hispanic Caucasian men in the seven cycles (P_linear trend _< 0.0001); the slope for this group was 1.03 (P-value = 0.04). In women, the age-adjusted OA prevalence of Non-Hispanic Caucasians increased from 11.95% (95% CI 10.23–13.68%) to 20.76% (95% CI 18.47–23.06%) in 2005–2014, and then remained approximately 18.5% from 2015 to 2018. OA’s prevalence among women in Hispanic, Non-Hispanic African American, and Non-Hispanic Other groups increased during 2005–2018. We observed a significant linear trend in all race/ethnicity groups except Non-Hispanic Other groups (all P_linear trend _≤ 0.02), and no apparent change in OA prevalence over time (slope_Non-Hispanic Caucasian _= 1.02, P-value = 0.04; slope_Hispanic _= 0.95, P-value = 0.005; slope_Non-Hispanic African American _= 0.40, P-value = 0.03).

### OA prevalence by SES in both genders

**Figure 3:**
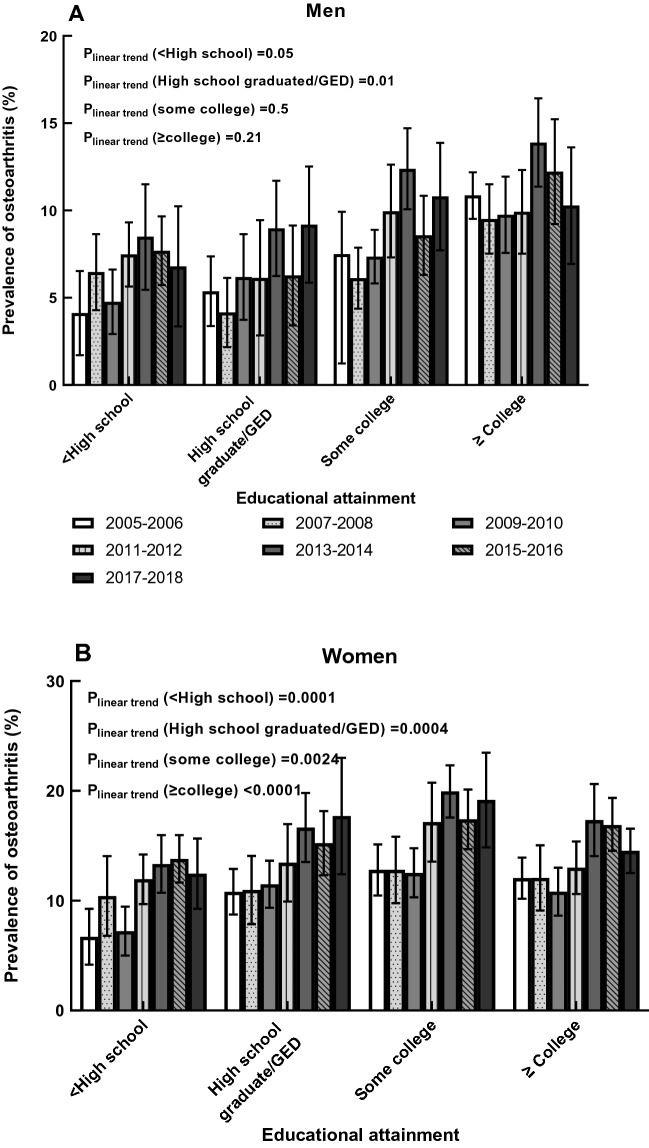
Age-adjusted prevalence of osteoarthritis by education level in men and women, 2005–2006 through 2017–2018.

The pattern of the age-adjusted prevalence of self-reported OA, stratified by education attainment in both men and women, is presented in Fig. 3. For men, the highest education level (≥ college) had a higher prevalence than other groups (all P-values ≤ 0.01). However, significant increasing linear trend were only observed among men with high school diploma/GED (P_linear trend _= 0.01) in 2005-2018 (slope = 0.65, P-value = 0.04). In men with high school diploma/GED, the adjusted OA prevalence increased in 2005–2018 from 5.37% (95% CI 3.38–7.37%) to 9.19% (5.86–12.52%). For women, age-adjusted OA prevalence among those with the lowest education attainment (less than high school) was lower than in other groups (all P-values ≤ 0.003). We observed significant linear trends among women in all education levels (all P_linear trend _< 0.04, slope_<high school _= 1.28, P-value = 0.02; slope_high school/GED _= 1.14, P-value = 0.004; slope_some college_ = 1.36, P-value = 0.02; slope_≥college_ = 0.88, P-value = 0.049). Specifically, age-adjusted OA prevalence among women with a high school diploma/GED kept increasing during the seven survey cycles, from 10.81% (95% CI 8.74–12.89%) to 17.70% (95% CI 12.40–23.00%).

**Figure 4:**
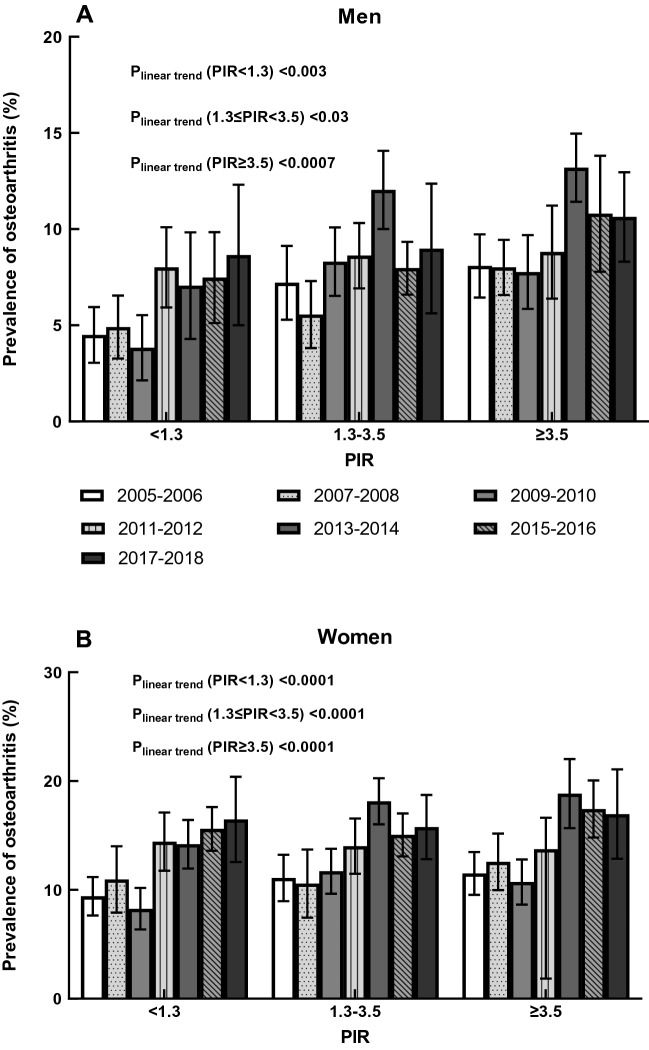
Age-adjusted prevalence of osteoarthritis by poverty income ratio level in men and women, 2005–2006 through 2017–2018.

The age-adjusted prevalence of self-reported OA by PIR for men and women is shown in Fig. 4. The men with the lowest family income (PIR < 1.3) had a lower age-adjusted OA prevalence among the two PIR groups (both P-values ≤ 0.0003). The age-adjusted prevalence of OA in this group increased from 4.51% (95% CI 3.51–5.51%) to 7.21% (95% CI 5.47–8.95%) in 2005–2012, then remained stable at around 7.5% in the last three survey cycles. Significant linear trends were observed among men with all PIR levels (all P_linear trend _< 0.03; slope_PIR<1.3 _= 0.71, P-value = 0.03; slope_1.3≤PIR<3.5_ = 0.77, P-value = 0.01; slope_PIR≥3.5_ = 0.53, P-value = 0.03). Women with the lowest PIR (PIR < 1.3) had a significantly lower age-adjusted prevalence of OA than those people with the highest PIR (P-value = 0.01). During the seven survey cycles, the prevalence fluctuated for women with the lowest family income, but the overall pattern increased. Significant linear trends were observed in all the three PIR groups among women (all P_linear trend _< 0.0001; slope_PIR<1.3_ = 1.33, P-value = 0.01; slope_1.3≤PIR<3.5_ = 1.06, P-value = 0.03; slope_PIR≥3.5_ = 1.24, P-value = 0.02).

### OA prevalence by weight, smoking, and physical activity status in both genders

The gender- and race-specific OA prevalence trends by weight, smoking, and physical activity status are presented in Supplementary Tables 2, 3 and 4. The trend of OA prevalence was similar among people with different weight, smoking, and physical activity status. For example, in obese people, significant linear trends were observed among Non-Hispanic Caucasian men (P_linear trend _< 0.0001) and women from Hispanic, Non-Hispanic Caucasian, and Non-Hispanic African American groups (P_linear trend_ ≤ 0.004). Among non-obese people, significant linear trends in OA prevalence were observed in Non-Hispanic Caucasian men (P_linear trend _= 0.012) and women from Hispanic, Non-Hispanic Caucasian, and Non-Hispanic African American groups (P_linear trend_ ≤ 0.0003).

### Multivariable-adjusted OA prevalence

The odds ratio from age-adjusted and multiple adjusted models by gender are shown in Table 2. For men, relative to 2005–2006, the positive associations were observed between age-adjusted OA prevalence and three survey cycles, including 2013–2014 (OR, 1.78; 95% CI 1.42–2.24), 2015–2016 (OR, 1.36; 95% CI 1.03–1.79), and 2017–2018 (OR, 1.46; 95% CI 1.05–2.02). The linear trend of OA prevalence in men across survey cycles was significant (P_linear trend_ = 0.0012). For women, the positive associations were found between age-adjusted OA prevalence and the last four survey cycles, the ORs were 1.41 (95% CI 1.11–1.80) in 2011–2012, 1.88 (95% CI 1.52–2.32) in 2013–2014, 1.70 (95% CI 1.39–2.08) in 2015–2016, and 1.76 (95% CI 1.31–2.35) in 2017–2018, respectively. The linear trend of OA prevalence in women across survey cycles was significant as well (P_linear trend_ = 0.0002). Additionally, we observed a significant linear trend of OA prevalence in men (P_linear trend_ = 0.009) and women (P_linear trend_ < 0.0001) after adjusting for age, race, educational attainment, PIR, weight status, smoking status, and physical activity.

**Table 2:** Adjusted association between survey cycle and prevalence of osteoarthritis by gender, 2005–2006 through 2017–2018.

Survey cycle	Sample size	Odds Ratios (95% CI)	
		Adjusted for age	Adjusted for age, race/ethnicity, educational attainment, PIR, obesity, smoking, and physical activity
**Men**			
2005–2006	2133	1 (reference)	1 (reference)
2007–2008	2499	0.90 (0.68–1.20)	0.91 (0.69–1.19)
2009–2010	2614	1.03 (0.78–1.34)	1.01 (0.77–1.32)
2011–2012	2359	1.27 (0.97–1.66)	1.25 (0.96–1.64)
2013–2014	2433	1.78 (1.42–2.24)	1.76 (1.40–2.20)
2015–2016	2328	1.36 (1.03–1.79)	1.31 (0.99–1.74)
2017–2018	2168	1.46 (1.05–2.02)	1.43 (1.04–1.97)
**Women**			
2005–2006	2326	1 (reference)	1 (reference)
2007–2008	2585	1.08 (0.77–1.51)	1.10 (0.79–1.52)
2009–2010	2785	0.98 (0.78–1.23)	0.97 (0.78–1.21)
2011–2012	2442	1.41 (1.11–1.80)	1.43 (1.13–1.82)
2013–2014	2661	1.88 (1.52–2.32)	1.86 (1.49–2.32)
2015–2016	2518	1.70 (1.39–2.08)	1.70 (1.39–2.07)
2017–2018	2320	1.76 (1.31–2.35)	1.70 (1.31–2.38)

*PIR* poverty income ratio.

## Discussion

In this study, with data from a nationally representative sample of US residents in noninstitutionalized populations, we found that women had a higher age-adjusted OA prevalence than men. In addition, significant linear trends and positive slope values of both genders indicate that the OA prevalence increased during 2005–2018. The increasing linear trend in OA prevalence in both genders still remained significant, even after additional adjustments were made in race/ethnicity, educational attainment, and PIR. Moreover, we found statistically significant linear trends in age-adjusted OA prevalence in Non-Hispanic Caucasian and Non-Hispanic African Americans of both genders and in Hispanic women. However, people with lower SES/educational attainment and low PIR reported a lower age-adjusted OA prevalence than people with higher SES. Also, we observed significant linear trends of OA prevalence in most SES subgroups for both genders (all P_linear trend_ ≤ 0.04).

The observed trends in OA prevalence among US adults during 2005–2018 were consistent with the study conducted by Dr. Park, which found that the age-adjusted prevalence of OA increased during 1999–2014^7^. Since obesity is a prominent risk factor for OA^19^, the increasing prevalence of this condition among adults in the US might contribute to the rising age-adjusted OA prevalence trend^28^. In our study, the percentage of obesity in both genders increased during 2005–2018, corresponding to the observed increasing OA prevalence trend. Furthermore, our findings regarding a higher prevalence of OA in women than in men also correspond to a prior meta-analysis study which found that women are generally at a higher risk of OA than men^29^. Joint space narrowing (JSN) is attributed to the loss of articular cartilage and leads to OA^30^, and women typically have a significantly more progressive decline in joint space than men^31^. Thus, the gender difference of JSN might partially explain the difference in OA prevalence between men and women. In both men and women, the significant linear trend in OA prevalence still exists after multiple adjustments for age, race/ethnicity, educational attainment, PIR, obesity, smoking, and physical activity. Apparently, changes in the distribution of these risk factors cannot fully explain the trend of OA prevalence over the years. In a study by Dr. Dillon et al. in 1991–1994, Non-Hispanic African Americans were reported to have a higher prevalence of knee OA than Non-Hispanic Caucasians. African Americans were more likely to have tibiofemoral joint (part of the knee) OA than Caucasians^32^, thus indicating that African Americans had a higher knee OA prevalence than Caucasians. However, in the present study, NHANES did not have information regarding that region of OA. Because our analysis used self-reported OAs, which includes OA in any joints, we could only analyze the prevalence of self-reported OA in any joints. Therefore, our results are different from Dr. Dillon’s observations. Caucasians were more likely to have OA on the spine^33^, hand, and other regions than African Americans^34^. In the present study, Caucasians had a higher OA prevalence than African Americans. Notably, we found that age-adjusted OA prevalence was lower among people with disadvantaged SES than people with higher SES. These findings were partially consistent with Dr. Park’s findings that OA was more prevalent in older Non-Hispanic Caucasian women with high family income or a college degree^7^. SES is an important determinant of access to healthcare^35^. People with higher SES are more likely to have better insurance coverage for accessing healthcare professionals and will presumably obtain more accurate diagnoses than those with low SES. In the current study, individuals with low SES might lack access to adequate healthcare for OA diagnosis, leading to lower self-reported OA prevalence in that particular group.

There are several limitations to this study. First, self-report data of doctor diagnosis were used to define OA in this study because radiographic data were unavailability in NHANES. Also, recall bias possibly impacts the accuracy of prevalence estimates. However, the CDC recommends using self-reported, doctor-diagnosed arthritis as the case definition in estimating the prevalence of arthritis^36^. Studies^28^ have proven the validity and reliability of such self-reported data. Second, a small percentage of NHANES participants lack valid information about educational attainment and thus were not eligible for the analysis. In the current study, 0.1% and 9.1% of eligible subjects lacked information about education level and family income, respectively, thus possibly leading to a biased estimate. Third, non-response bias is always a concern in NHANES data, as response rates have declined in federal surveys since 2000^37^. The decline in response rates could have a different impact on OA’s estimated prevalence accuracy across the different survey cycles we studied. However, the sample weights of NHANES have accounted for non-response in the analysis. Therefore, these limitations are unlikely to have altered the trends of OA prevalence we observed.

## Conclusion

In summary, an increasing trend in OA’s age-adjusted prevalence was observed among US men and women during 2005–2018. Non-Hispanic Caucasian and Non-Hispanic African Americans had significantly increasing linear trends in OA prevalence in both genders. People with disadvantaged SES had a lower prevalence of OA. Considering the work limitations and economic burden caused by OA, our findings may be informative in developing related policies to reduce disease development among the population and reduce related risk factors. Our results of OA disparities suggest a need to increase public and health system awareness of OA, especially in Non-Hispanic Caucasian and Non-Hispanic African Americans. Additional research is warranted to further explain the increasing trend in OA prevalence in different races/ethnicities and SES groups in order to more accurately determine the most effective strategies for preventing OA and reducing such glaring disparities.

## Supplementary Information

Supplementary Tables.
