# Supplementary material for: Trends and disparities in osteoarthritis prevalence among US adults, 2005–2018
Source: Sci Rep. 2021 Nov 8;11:21845. doi: 10.1038/s41598-021-01339-7 (PMC8576014; doi:10.1038/s41598-021-01339-7)
Supplement: Supplementary file 1 — Supplementary Tables. [file 41598_2021_1339_MOESM1_ESM.docx]

**Supplementary Table 1** Risk factors Related to Osteoarthritis of Participants from Seven National Health and Nutrition Examination Surveys 2005-2006 through 2017-2018

|  | 2005-2006  (n=4,459) | 2007-2008  (n=5,084) | 2009-2010  (n=5,399) | 2011-2012  (n=4,801) | 2013-2014  (n=5,094) | 2015-2016  (n=4,846) | 2017-2018  (n=4,488) |
| --- | --- | --- | --- | --- | --- | --- | --- |
| Men, No. (Weighted %) |  | | | | | | |
| Obesity^a^ | 680 (33.00) | 841(32.22) | 938 (36.00) | 757 (33.56) | 806 (35.15) | 847 (38.62) | 865 (43.32) |
| Current smoker^b^ | 560 (27.50) | 647 (25.94) | 628 (21.90) | 599 (23.61) | 561 (21.06) | 530 (20.09) | 477 (19.46) |
| Physical inactive^c^ | 170 (6.13) | 564 (14.56) | 604 (17.48) | 469 (16.76) | 496 (18.72) | 493 (15.96) | 449 (15.67) |
| Women, No. (Weighted %) |  | | | | | | |
| Obesity^a^ | 901 (35.90) | 1020 (35.02) | 1140 (36.49) | 980 (36.60) | 1122 (40.68) | 1122 (41.43) | 1024 (42.97) |
| Current smoker^b^ | 413 (20.49) | 503 (19.90) | 551 (19.09) | 369 (16.03) | 491 (19.32) | 381 (15.94) | 340 (15.53) |
| Physical inactive^c^ | 274 (10.06) | 899 (27.69) | 895 (28.26) | 717 (24.89) | 811 (28.65) | 776 (25.20) | 692 (25.00) |

^a^Obesity: people with body mass index ≥30.

^b^Current smoker: People had smoked at least 100 cigarettes during their lifetime and reported smoking either every day or some days.

^c^Physical inactive: people who were sedentary or only did basic activities, which refers to light-intensity activities like standing and walking slowly

|  | 2005-2006  (N=4,278) | 2007-2008  (N=4,876) | 2009-2010  (N=5,105) | 2011-2012  (N=4,009) | 2013-2014  (N=4,360) | 2015-2016  (N=4,104) | 2017-2018  (N=3,610) | P _linear trend_ |  |
| --- | --- | --- | --- | --- | --- | --- | --- | --- | --- |
| Obese |  | | | | | | | | |
| Men |  | | | | | | | | |
| Hispanic^a^ | 6.63  (3.77-9.49) | 6.83  (4.31-9.35) | 7.38  (5.62-9.15) | 8.38  (5.0811.67) | 6.88  (3.05-10.71) | 8.91  (6.61-11.21) | 5.24  (2.44-8.40) | 0.99 |  |
| NH-Caucasian | 10.26  (8.31-12.20) | 5.85  (3.17-8.54) | 8.76  (6.15-11.37) | 9.99  (7.07-12.90) | 17.34  (14.69-19.99) | 13.60  (9.89-17.31) | 13.13  (9.20-17.05) | 0.0001 |  |
| NH-African American | 5.72  (3.05-8.40) | 5.33  (2.70-7.97) | 6.61  (3.26-9.96) | 6.12  (2.65-9.59) | 6.86  (1.68-12.03) | 8.32  (5.17-11.47) | 7.57  (3.75-11.39) | 0.19 |  |
| Women |  | | | | | | | | |
| Hispanic^a^ | 6.01  (3.58-8.44) | 6.98  (4.47-9.49) | 6.56  (3.99-9.14) | 12.05  (8.87-15.23) | 11.47  (8.87-14.07) | 9.40  (7.32-11.48) | 9.76  (6.19-13.33) | 0.004 |  |
| NH-Caucasian | 15.57  (12.70-18.44) | 11.94  (8.71-15.17) | 13.88  (16.68-17.08) | 19.60  (14.88-24.31) | 27.86  (22.70-33.02) | 20.03  (16.96-23.10) | 23.39  (17.48-29.31) | 0.0001 |  |
| NH-African American | 7.37  (4.12-10.62) | 7.76  (5.12-10.40) | 8.06  (4.56-11.56) | 12.07  (9.47-14.67) | 13.72  (10.15-17.29) | 11.08  (8.10-14.07) | 14.18  (10.96-17.40) | 0.0004 |  |
| Non-Obese |  | | | | | | | | |
| Men |  | | | | | | | |  |
| Hispanic^a^ | 1.71  (0.38-3.05) | 2.38  (0.66-4.10) | 2.65  (1.20-4.10) | 4.19  (2.09-6.29) | 3.69  (2.01-5.37) | 1.00  (0, 2.75) | 5.62  (2.95-8.29) | 0.06 |  |
| NH-Caucasian | 6.36  (4.78-7.95) | 7.78  (6.24-9.32) | 7.61  (5.29-9.93) | 9.66  (7.33-11.99) | 12.04  (9.69-14.38) | 8.29  (5.76-10.82) | 9.34  (6.66-12.02) | 0.012 |  |
| NH-African American | 3.52  (1.40-5.64) | 2.16  (0.10-4.23) | 3.41  (1.88-4.93) | 3.75  (1.59-5.91) | 6.08  (3.53-8.63) | 4.05  (1.87-6.23) | 4.62  (1.68-7.56) | 0.14 |  |
| Women |  | | | | | | | |  |
| Hispanic^a^ | 3.94  (2.32-5.57) | 6.34  (4.39-8.29) | 6.56  (3.99-9.14) | 7.01  (5.21-8.81) | 6.34  (4.73-7.96) | 8.40 (6.23-10.56) | 10.15  (6.94-13.37) | 0.0001 |  |
| NH-Caucasian | 10.03  (7.68-12.38) | 13.18  (9.71-16.65) | 13.88  (16.68-17.08) | 13.92  (11.43-16.41) | 16.13  (13.66-18.60) | 17.13  (14.98-19.27) | 15.31  (11.87-18.76) | 0.0003 |  |
| NH-African American | 5.04  (2.84-7.24) | 5.40  (3.02-7.79) | 8.06  (4.56-11.56) | 5.86  (3.26-8.46) | 8.01  (4.67-11.36) | 10.73  (8.08-13.38) | 8.43  (5.51-11.36) | 0.0001 |  |

**Supplementary Table 2** Age-adjusted Prevalence of Osteoarthritis in Adults with by Weight Status during National Health and Nutrition Examination Surveys 2005-2006 through 2017-2018*

* The results of NH-Other are not shown because some estimates are potentially unreliable due to the limited sample size.

Abbreviations: NH-Caucasian=Non-Hispanic Caucasian; NH-African American=Non-Hispanic African American

^a^ Hispanic includes Mexican American and other Hispanic

**Supplementary Table 3** Age-adjusted Prevalence of Osteoarthritis in Adults by Smoking Status during National Health and Nutrition Examination Surveys 2005-2006 through 2017-2018*

|  | 2005-2006  (N=4,278) | 2007-2008  (N=4,876) | 2009-2010  (N=5,105) | | 2011-2012  (N=4,009) | | 2013-2014  (N=4,360) | | 2015-2016  (N=4,104) | | 2017-2018  (N=3,610) | | P _linear trend_ | |
| --- | --- | --- | --- | --- | --- | --- | --- | --- | --- | --- | --- | --- | --- | --- |
| Current smokers |  | | | | | | | | | | | | | |
| Men |  | | | | | | | | | | | | | |
| Hispanic^a^ | 3.29  (0.69-7.28) | 1.58  (0.90-4.05) | | 2.77  (0.31-5.22) | | 6.16  (1.14-11.17) | | 4.88  (1.71-8.05) | | 4.40  (0.90-7.89) | | 6.02  (1.90-10.14) | | 0.1 |
| NH-Caucasian | 5.24  (3.08-7.40) | 6.40  (3.84-8.96) | | 5.68  (2.93-8.42) | | 8.08  (4.31-11.85) | | 12.23  (7.21-17.25) | | 11.76  (8.20-15.32) | | 15.04  (8.20-21.87) | | <0.0001 |
| NH-African American | 3.55  (0.93-6.17) | 1.50  (0.48-3.48) | | 0.45  (0-2.76) | | 6.03  (0.36-11.70) | | 5.56  (1.31-9.82) | | 5.06  (1.00-9.12) | | 6.27  (1.22-11.32) | | 0.05 |
| Women |  | | | | | | | | | | | | | |
| Hispanic^a^ | 7.15  (2.80-11.49) | 8.72  (5.77-11.66) | | 11.05  (6.71-15.40) | | 14.13  (6.50-21.75) | | 13.08  (6.76-19.39) | | 12.40  (8.64-16.15) | | 12.37  (4.56-20.17) | | 0.06 |
| NH-Caucasian | 7.98  (4.73-11.23) | 12.43  (8.78-16.09) | | 12.85  (9.29-16.42) | | 14.94  (9.05-20.83) | | 19.32  (13.03-25.61) | | 18.65  (13.43-23.88) | | 20.28  (13.16-27.39) | | <0.0001 |
| NH-African American | 4.24  (0.95-7353) | 5.48  (2.42-8.55) | | 5.58  (2.59-8.58) | | 10.84  (5.75-15.94) | | 10.60  (6.45-14.76) | | 9.91  (5.97-13.85) | | 9.46  (3.25-15.66) | | 0.0084 |
| Former smokers/Non-smokers |  | | | | | | | | | | | | | |
| Men |  | | | | | | | | | | | | | |
| Hispanic^a^ | 3.31  (1.72-4.92) | 4.43  (2.54-6.32) | | 4.98  (3.67-6.28) | | 5.93  (4.04-7.83) | | 4.93  (2.17-6.05) | | 4.10  (2.17-6.05) | | 4.99  (2.44-7.55) | | 0.43 |
| NH-Caucasian | 8.36  (6.55-10.18) | 7.15  (5.88-8.42) | | 8.39  (6.18-10.60) | | 9.91  (7.46-12.35) | | 14.03  (12.03-16.03) | | 9.96  (7.06-12.86) | | 9.99  (7.51-12.48) | | 0.006 |
| NH-African American | 4.55  (2.37-6.73) | 3.82  (1.20-6.44) | | 6.29  (3.52-9.04) | | 4.73  (2.11-7.35) | | 6.66  (3.25-10.07) | | 5.64  (3.40-7.88) | | 6.06  (2.78-9.34) | | 0.25 |
| Women |  | | | | | | | | | | | | | |
| Hispanic^a^ | 4.28  (3.08-5.48) | 6.35  (4.16-8.55) | | 4.55  (2.91-6.19) | | 8.55  (7.14-9.97) | | 8.33  (6.31-10.35) | | 8.69  (6.61-10.77) | | 9.73  (7.14-12.32) | | <0.0001 |
| NH-Caucasian | 12.67  (10.42-14.90) | 12.40  (9.36-15.45) | | 11.30  (9.52-13.07) | | 15.86  (13.26-18.46) | | 20.76  (17.37-24.15) | | 17.87  (15.68-20.06) | | 18.06  (14.05-22.07) | | <0.0001 |
| NH-African American | 6.74  (4.51-8.94) | 6.97  (4.89-9.07) | | 7.09  (4.68-9.50) | | 8.59  (5.88-11.29) | | 11.29  (7.86-14.72) | | 11.46  (9.77-13.14) | | 12.03  (9.15-14.92) | | <0.0001 |

*The results of NH-Other are not shown because some estimates are potentially unreliable due to the limited sample size.

Abbreviations: NH-Caucasian=Non-Hispanic Caucasian; NH-African American=Non-Hispanic African American

^a^ Hispanic includes Mexican American and other Hispanic

**Supplementary Table 4** Age-adjusted Prevalence of Osteoarthritis in Adults by Physical Active Status during National Health and Nutrition Examination Surveys 2005-2006 through 2017-2018*

|  | 2005-2006  (N=4,278) | 2007-2008  (N=4,876) | 2009-2010  (N=5,105) | 2011-2012  (N=4,009) | 2013-2014  (N=4,360) | 2015-2016  (N=4,104) | 2017-2018  (N=3,610) | P _linear trend_ |
| --- | --- | --- | --- | --- | --- | --- | --- | --- |
| Physically active |  | | | | | | | |
| Men |  | | | | | | | |
| Hispanic^a^ | 3.25  (1.84-4.67) | 3.49  (1.90-5.08) | 4.60  (3.41-5.79) | 5.42  (3.23-7.62) | 4.02  (1.54-6.49) | 4.41  (2.54-6.28) | 5.13  (3.06-7.20) | 0.14 |
| NH-Caucasian | 7.82  (6.223-9.41) | 7.53  (6.28-8.77) | 8.56  (6.94-10.19) | 9.81  (7.22-12.39) | 13.05  (11.11-14.99) | 10.02  (7.63-12.40) | 11.16  (8.22-14.10) | 0.0009 |
| NH-African American | 4.33  (2.39-6.28) | 3.84  (2.15-5.53) | 4.57  (2.83-6.30) | 5.03  (2.69-7.37) | 5.26  (5.53-7.89) | 6.20  (3.86-8.53) | 6.62  (3.92-9.43) | 0.05 |
| Women |  | | | | | | | |
| Hispanic^a^ | 4.53  (3.01-6.04) | 7.03  (4.66-9.41) | 5.29  (3.47-7.11) | 7.86  (5.21-10.51) | 8.18  (6.28-10.07) | 9.12  (6.80-11.44) | 9.88  (6.58-13.19) | 0.0001 |
| NH-Caucasian | 11.59  (9.38-13.80) | 13.55  (10.50-16.60) | 11.74  (10.08-13.40) | 14.73  (12.08-17.37) | 19.70  (17.10-22.30) | 17.21  (14.97-19.47) | 18.40  (14.62-22.18) | 0.0001 |
| NH-African American | 6.12  (4.22-8.02) | 7.89  (5.51-10.29) | 5.76  (3.87-7.65) | 7.92  (5.96-9.99) | 9.93  (7.08-12.79) | 11.42  (9.15-13.69) | 11.89  (9.05-14.73) | <0.0001 |
| Physically inactive |  | | | | | | | |
| Men |  | | | | | | | |
| Hispanic^a^ | 1.82  (0-7.18) | 5.97  (3.05-8.88) | 3.26  (0.64-5.88) | 7.14  (2.34-11.94) | 9.58  (4.43-14.72) | 4.01  (0.25-8.26) | 5.98  (1.50-10.47) | 0.21 |
| NH-Caucasian | 7.15  (0.63-13.66) | 5.44  (2.51-8.36) | 5.00  (0.53-9.47) | 9.87  (5.40-14.33) | 17.80  (13.45-22.16) | 12.34  (9.73-14.96) | 10.18  (5.17-15.20) | 0.009 |
| NH-African American | 2.32  (0-8.67) | 2.38  (0-5.47) | 4.22  (0.22-8.22) | 4.92  (0.91-8.93) | 11.99  (4.90-19.08) | 3.51  (0-7.16) | 4.57  (1.04-10.19) | 0.24 |
| Women |  | | | | | | | |
| Hispanic^a^ | 4.91  (0.54-10.37) | 5.76  (3.36-8.17) | 5.78  (3.56-8.00) | 11.84  (8.51-15.18) | 9.51  (5.56-13.45) | 8.31  (5.90-10.72) | 10.04  (7.05-13.04) | 0.02 |
| NH-Caucasian | 15.94  (10.39-21.49) | 10.94  (7.00-14.87) | 13.22  (9.53-16.91) | 20.27  (14.74-25.80) | 23.44  (19.71-27.17) | 22.35  (16.80-27.89) | 19.95  (14.64-25.25) | 0.001 |
| NH-African American | 7.58  (2.08-13.08) | 4.34  (1.95-6.73) | 8.91  (5.17-12.64) | 11.79  (8.30-15.27) | 14.09  (8.89-19.29) | 9.98  (6.71-13.25) | 10.80  1.(6.61-14.99) | 0.03 |

*The results of NH-Other are not shown because some estimates are potentially unreliable due to the limited sample size.

Abbreviations: NH-Caucasian=Non-Hispanic Caucasian; NH-African American=Non-Hispanic African American

^a^ Hispanic includes Mexican American and other Hispanic
